# Development and validation of predictive model for emesis in cervical cancer patients receiving concurrent chemoradiotherapy based on multi-institutional retrospective study

**DOI:** 10.1038/s41598-025-21494-5

**Published:** 2025-10-27

**Authors:** Kensuke Yoshida, Hajime Morita, Masaki Nakai, Yusuke Kawamura, Takuma Matsumoto, Yoshinobu Gohara, Naoto Hoshino, Naoya Tonomura, Manami Banba, Ayako Yamaguchi, Masaki Tachibana, Tomoki Fukushima, Hiroki Hosokawa, Takuya Mura, Tsuyoshi Yabuki, Kyongsun Pak, Shinichi Watanabe, Anna Kiyomi, Noriaki Hidaka, Chie Saito, Takahiro Kobayashi, Tomokazu Shoji, Motoko Kaneko, Masayoshi Koga, Tomoya Nozaki, Munetoshi Sugiura

**Affiliations:** 1 Department of Drug Safety and Risk Management, School of Pharmacy, Tokyo University of Pharmacy and Life Sciences, Horinouchi, Hachioji, Tokyo, 1432-1192-0392 Japan; 2 Division of Pharmacy, St. Marianna University Hospital, Kawasaki, Japan; 3 Division of Pharmacy, Ehime University Hospital, Ehime, Toon, Japan; 4 Division of Hospital Pharmacy, Hiroshima City Hiroshima Citizens Hospital, Hiroshima, Japan; 5 Division of Hospital Pharmacy, NHO Shikoku Cancer Center, Ehime, Matsuyama, Japan; 6 Department of Pharmacy, Saga University Hospital, Saga, Japan; 7 Division of Hospital Pharmacy, Niigata University Medical and Dental Hospital, Niigata, Japan; 8 Division of Hospital Pharmacy, Tokyo Metropolitan Tama Medical Center, Fuchu, Japan; 9 Division of Hospital Pharmacy, Aomori Prefectural Central Hospital, Aomori, Japan; 10 Department of Pharmacy, Yamagata University Hospital, Yamagata, Japan; 11 Division of Pharmacy, Nagaoka Red Cross Hospital, Nagaoka, Japan; 12 Department of Pharmacy, University of Yamanashi Hospital, Chuo, Japan; 13 Division of Hospital Pharmacy, Nagaoka Chuo General Hospital, Nagaoka, Japan; 14 Division of Hospital Pharmacy, National Hospital Organization Iwakuni Clinical Center, Iwakuni, Japan; 15 Division of Pharmacy, Uonuma Kikan Hospital, Minamiuonuma, Japan; 16 Division of Biostatistics, Center for Clinical Research, National Center for Child Health and Development, Setagaya, Japan; 17 Department of Clinical Pharmacy, College of Pharmaceutical Sciences, Matsuyama University, Matsuyama, Japan

**Keywords:** Chemotherapy-induced nausea and vomiting, Predictive model, Chemoradiotherapy, Multi-institutional, Retrospective cohort study, Cancer, Cancer models, Cancer prevention, Cancer therapy, Gynaecological cancer

## Abstract

Chemotherapy-induced nausea and vomiting (CINV) remains a significant barrier to treatment adherence and quality of life in cervical cancer patients receiving chemoradiotherapy. No validated predictive models exist to assess CINV risk in this population. We aimed to develop and temporal validate a predictive model for CINV incidence in cervical cancer patients receiving concurrent chemoradiotherapy (CCRT). This multi-institutional, retrospective cohort study analyzed 921 patients who received CCRT with weekly cisplatin (40 mg/m^2^) between January 2016 and March 2024. Candidate predictors were selected through expert consultations and literature reviews. A multivariate logistic regression model was developed using training data, and the model with the highest receiver operating characteristic-area under the curve (ROC-AUC) was tested using validation data. The model (age, smoking history, total radiation dose, chemotherapy history, 5-hydroxytryptamine 3 receptor antagonist use, cancer stage) showed good discrimination. In the training dataset, the model achieved an ROC-AUC of 0.772 (95% confidence interval [CI], 0.717–0.827). In the validation dataset, the model showed high discriminative ability (ROC-AUC, 0.808; 95%CI, 0.763–0.853) and good calibration (intraclass correlation coefficient, 0.826; *p* < 0.001). We developed and validated a clinically useful CINV prediction model for cervical cancer patients receiving CCRT. This tool may individualize antiemetic strategies and improve care.

**Supplementary Information:**

The online version contains supplementary material available at 10.1038/s41598-025-21494-5.

## Introduction

Chemotherapy-induced nausea and vomiting (CINV) is a significant adverse effect in patients receiving cancer treatment, leading to appetite loss, malnutrition, and psychological distress^1,2^. These symptoms severely impair patients’ quality of life (QOL) and negatively affect treatment adherence and continuation^3^. Among the highly emetogenic chemotherapy (HEC) agents, cisplatin induces vomiting in > 90% of patients^4^. Additionally, female patients are at a higher risk of CINV than male patients, and a Japanese multicenter phase II prospective study demonstrated that approximately 30% of patients remained emesis-free during treatment^5^.

Cervical cancer is a malignancy that exclusively affects women, a population inherently at increased risk of CINV^6,7^. Furthermore, in Japan, the emetogenic risk classification of chemoradiotherapy for cervical cancer was recently revised from moderately emetogenic chemotherapy to HEC, reflecting its increased potential to cause severe nausea and vomiting^8^.This reclassification underscores the need for enhanced antiemetic strategies tailored for this patient population. Accordingly, the latest clinical guidelines recommend the use of olanzapine to prevent CINV.

Despite the well-recognized burden of CINV in patients with cervical cancer, no predictive model has been established for the incidence of CINV specific to chemoradiotherapy in this population in Japan. Several studies have previously identified risk factors for CINV, including age, female sex, smoking status, radiation dose, history of chemotherapy, use of serotonin 5-hydroxytryptamine 3 (5-HT3) receptor antagonists, and cancer stage^9–16^. These findings underscore the importance of integrating clinically relevant predictors when developing a robust predictive model for CINV. Furthermore, radiation-induced nausea and vomiting (RINV) has been reported particularly in cases involving abdominal or pelvic irradiation^17^, underscoring the importance of considering radiotherapy itself as a contributing factor to emesis in the treatment of cervical cancer. Given the recent guideline updates and the high emetogenic risk associated with chemoradiotherapy for cervical cancer^18^, developing a predictive model for CINV risk is essential for optimizing supportive care. A validated model would enable the early identification of high-risk patients and facilitate the implementation of personalized antiemetic interventions to improve QOL and treatment adherence.

This study aimed to develop and validate a predictive model for the incidence of CINV in patients receiving chemoradiotherapy for cervical cancer. By integrating this model into clinical practice, we aimed to establish a systematic framework for risk assessment and intervention, thereby minimizing the adverse effects of CINV on patient outcomes.

## Methods

### Study population

This retrospective cohort study included patients who received concurrent chemoradiotherapy (CCRT) between January 2016 and March 2024. This multicenter retrospective study included data from 14 participating hospitals across Japan, including university hospitals, cancer centers, and general hospitals. The full list of institutions and their locations is provided in the Author Affiliations section. All patients received CCRT with cisplatin regimen administered weekly at a dose of 40 mg/m^2^. Patients were excluded if they met any of the following criteria: those who presented with nausea at the initiation of CCRT; those with identifiable risk factors for nausea unrelated to CCRT, such as gastroesophageal reflux disease, peptic ulcer, or pregnancy; those who started CCRT with a dose reduction from the first cycle; those who required a change in the treatment regimen for reasons other than nausea after completing the first cycle of CCRT; and those who participated in other clinical trials or interventional studies during the study period. The presence of unrelated nausea risk factors was determined based on pre-existing diagnoses documented in the patients’ medical records. These assessments were made by site pharmacists following standardized criteria.

### Data collection and variables

Data regarding the following variables were collected: (1) patient factors (age, body mass index, smoking history, alcohol consumption history, use of corticosteroids, use of immunosuppressive agents, prior chemotherapy, opioid use, total radiation dose, cancer stage), (2) hematological values (albumin levels, white blood cell counts, neutrophil counts, platelet counts, and total protein), and (3) vomiting severity status. Patients with missing values in any of the candidate predictor variables were excluded from the analysis to ensure consistency and comparability across models. No imputation was performed for missing data. The total radiation dose was recorded in Gray (Gy), which is standard in Japanese clinical practice. Chemotherapy history included any prior systemic chemotherapy, including neoadjuvant chemotherapy. Cancer staging was based on the International Federation of Gynecology and Obstetrics (FIGO) classification, which is routinely used in gynecologic oncology in Japan.

Patient factors and hematological data were collected at the initiation of chemotherapy. The incidence and severity of vomiting were assessed using the Common Terminology Criteria for Adverse Events version 5.0. Grade 1 was defined as vomiting that did not require medical intervention. Grade 2 was defined as vomiting requiring outpatient intravenous fluid administration. Grade 3 was defined as vomiting necessitating enteral nutrition, total parenteral nutrition, or hospitalization. Vomiting was assessed across the full duration of CCRT and any episode of vomiting (grade 1 or higher) during this period was defined as CINV. Vomiting was defined as any event of grade 1 or higher according to CTCAE version 5.0 and was assessed across the full duration of CCRT (typically 6–8 weeks), thereby capturing both acute and delayed CINV. Data on vomiting were obtained from medical records and routinely documented CTCAE evaluations.

### Statistical analyses

Candidate variables were selected for the prediction model using a three-step process. First, we divided the dataset into two parts, one for model derivation (CCRT start date: from January 2016 to December 2019) and one for validation (CCRT start date: January 2020 to March 2024), and summarized each dataset. Second, we identified four primary baseline factors as initial candidate predictors: age, smoking history, total radiation dose, and chemotherapy history. To improve the predictive accuracy of the model, we incorporated three additional variables: 5-HT3—a serotonin blocker used for the prevention and treatment of nausea and vomiting caused by chemoradiotherapy, cancer stage, and olanzapine. The clinical relevance of 5-HT3 receptor antagonists in the prevention of CINV has been well established in prior literature. Combination antiemetic regimens incorporating 5-HT3 antagonists are standard for patients at high emetic risk^14,15^. While cancer stage was not directly analyzed as a predictor in that study, disease stage often influences treatment intensity and radiotherapy field, which in turn may affect the incidence of nausea and vomiting^16^. These variables were selected based on their established or hypothesized relevance to CINV. These predictors were determined through consultation with seven board-certified oncology pharmacists accredited by the Japanese Society of Pharmaceutical Health Care and Sciences. Multivariable logistic regression models were developed using all possible combinations of the seven candidate predictors in the derivation dataset, with the final set selected based on the combination that achieved the highest receiver operating characteristic-area under the curve (ROC-AUC). We estimated the CINV odds ratios (ORs) and 95% confidence intervals (CIs) for each candidate model, and the 200 times repeated 3-folds cross-validated the ROC-AUC and the 95% CI using the bootstrap method (repeated 2000 times) as an indicator of the prediction performance of the model. In addition, the Brier score was calculated to evaluate the prediction errors. The model with the maximum ROC-AUC was selected as the optimal scoring model. The final model was applied to the validation data, the ROC-AUC and 95% CI were calculated using the same method as the model derivation. In addition, the Brier score, calibration plot, and intraclass correlation coefficient were calculated. Model calibration was assessed using calibration plots that compared the predicted and observed CINV across the 20 strata. The Hosmer–Lemeshow goodness-of-fit test was used to evaluate the calibration performance. The final model was re-estimated using all data integrated from the derivation and validation data, and the ORs, ROC-AUCs, 95% CIs, and Brier scores were re-evaluated. The final model was re-estimated using the combined derivation and validation datasets to provide comprehensive summary statistics across the full study cohort. This re-estimated model is intended for descriptive purposes only and is not intended for immediate clinical implementation without further prospective validation. All statistical analyses were performed using R version 4.1.2 (The R Foundation; Indiana, US).

### Ethics declaration

This study was conducted in accordance with the principles of the Declaration of Helsinki and approved by the Research Ethics Committee of St. Marianna University, School of Medicine Hospital (approval number: 6560 [B67]).

## Results

### Patient characteristics

**Fig. 1:**
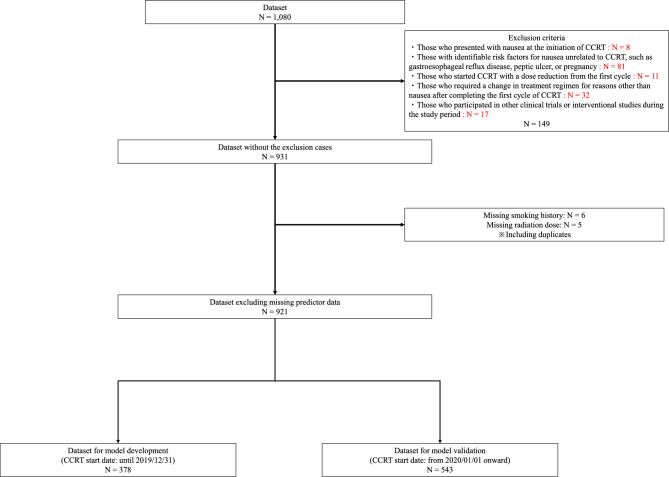
Flowchart of patient selection and dataset construction. Among the initially identified patients who received concurrent chemoradiotherapy (CCRT), those meeting predefined exclusion criteria, such as preexisting nausea, unrelated medical conditions, or participation in other clinical trials, were excluded. Subsequently, the dataset was divided into two groups based on the start date of CCRT: model development (patients who initiated CCRT on or before December 31, 2019) and temporal validation (patients who received CCRT on or after January 1, 2020). Patients with missing predictive variables were excluded from the final analyses. Patients with missing data for any predictor variables were excluded.

This retrospective cohort study included 921 patients who received CCRT (Fig. 1). Table 1 summarizes the baseline characteristics of the patients stratified based on the presence or absence of vomiting in both the training and validation datasets. In the training dataset, 106 patients experienced vomiting and 272 did not. In the validation dataset, 117 patients experienced vomiting and 426 did not. Although most baseline characteristics were similar between the two groups, the frequency of olanzapine use varied between datasets. In the training dataset, olanzapine was administered to 3.8% (four patients) of those with vomiting and 7.4% (20 patients) of those without vomiting. In contrast, the validation dataset showed higher overall usage, with 12.0% (14 patients) in the vomiting group and 11.3% (48 patients) in the non-vomiting group. The proportions of patients with stage I disease were 25.5% (27 patients) and 30.9% (84 patients) in the vomiting and non-vomiting groups of the training dataset, respectively, and 12.8% (15 patients) and 16.0% (68 patients) in the validation dataset, respectively. For stage II, the corresponding proportions were 43.4% and 41.2% in the training dataset and 24.8% and 32.2% in the validation dataset, respectively. Stage III patients accounted for 19.8% and 14.7% of the training dataset and 49.6% and 41.3% of the validation dataset, respectively, showing a relatively higher proportion in the vomiting group than that in the non-vomiting group. The stage IV distribution remained consistent at approximately 10–13% across all groups (Table 1).

**Table 1:** Patient characteristics.

Variable	Category	Training dataset		Validation dataset	
		CINV		CINV	
		Yes	No	Yes	No
N		106	272	117	426
Age	Mean ± SD	53.1 ± 12.0	57.6 ± 13.0	56.7 ± 12.3	56.5 ± 12.5
Body mass index	Median (IQR)	21.6 (19.5–25.0)	21.8 (19.4–23.9)	22.2 (19.3–25.0)	21.8 (19.2–25.3)
Smoking history	Yes N(%)	36 (34.0%)	107 (39.3%)	36 (30.8%)	167 (39.2%)
Alcohol consumption history	Yes N(%)	34 (32.1%)	92 (33.8%)	31 (26.5%)	139 (32.6%)
Use of corticosteroids	Yes N(%)	2 (1.9%)	2 (0.7%)	4 (3.4%)	13 (3.1%)
Use of immunosuppressive agents	Yes N(%)	1 (0.9%)	1 (0.4%)	0 (0.0%)	3 (0.7%)
Prior chemotherapy	Yes N(%)	14 (13.2%)	27 (9.9%)	7 (6.0%)	28 (6.6%)
Opioid use	Yes N(%)	19 (17.9%)	11 (4.0%)	15 (12.8%)	29 (6.8%)
Total radiation dose	Mean ± SD	54.1 ± 29.2	60.1 ± 18.1	44.8 ± 26.4	63.4 ± 17.5
Cancer stage	I N(%)	27 (25.5%)	84 (30.9%)	15 (12.8%)	68 (16.0%)
	II N(%)	46 (43.4%)	112 (41.2%)	29 (24.8%)	137 (32.2%)
	III N(%)	21 (19.8%)	40 (14.7%)	58 (49.6%)	176 (41.3%)
	IV N(%)	12 (11.3%)	36 (13.2%)	15 (12.8%)	44 (10.3%)
Albumin levels (g/dl)	Median (IQR)	4.0 (3.5—4.2)	3.9 (3.5—4.1)	3.9 (3.4—4.2)	3.9 (3.5–4.2)
White blood cell counts	Median (IQR)	5605.0 (4325.0—7377.5)	5800.0 (4400.0—7242.5)	5840.0 (4800.0—7900.0)	6500.0 (4900.0–8497.5)
Neutrophils counts	Median (IQR)	3500.0 (2560.0—5328.0)	3950.0 (2810.0—5137.0)	3710.0 (2950.0—6042.0)	4320.0 (3054.0–6006.0)
Platelet counts	Median (IQR)	28.0 (22.3—33.8)	27.2 (22.4—36.9)	27.3 (22.6—34.9)	28.8 (23.1–36.8)
Total protein	Median (IQR)	6.9 (6.6—7.3)	6.9 (6.4—7.3)	7.0 (6.5—7.4)	7.0 (6.5–7.4)
Olanzapine use	Yes N(%)	4 (3.8%)	20 (7.4%)	14 (12.0%)	48 (11.3%)
NK1 receptor antagonist use	Yes N(%)	39 (36.8%)	199 (73.2%)	48 (41.0%)	338 (79.3%)
5-HT3receptor antagonist use	Granisetron N(%)	56 (52.8%)	47 (17.3%)	54 (46.2%)	60 (14.1%)
	Palonosetron N(%)	50 (47.2%)	225 (82.7%)	63 (53.8%)	366 (85.9%)

CINV, chemotherapy-induced nausea and vomiting; SD, standard deviation; IQR, interquartile range.

### Predictive accuracy of the final model in the training datasets

Olanzapine was initially included as a candidate predictor based on clinical relevance. However, the model containing olanzapine did not achieve the highest ROC-AUC among all variable combinations. Given its low usage rate during the study period and its limited predictive utility in the current Japanese context, where guidelines recommend olanzapine for all patients receiving HEC, it was excluded from the final model. Including it could also reduce the model’s applicability across diverse settings. For transparency, the ROC-AUC, Brier score, adjusted ORs, and 95% CIs for the other models are provided in Supplementary Table S1. The model incorporating age, smoking history, total radiation dose, history of chemotherapy, 5-HT_3_ receptor antagonist use, and cancer stage demonstrated the highest predictive performance among all candidates. The cross-validated ROC-AUC was 0.772 (95% CI, 0.717–0.827), with a Brier score of 0.166, indicating good discriminative ability and calibration. In this model, 5-HT3 receptor antagonist use was strongly associated with a reduced risk of vomiting (OR, 0.13; 95% CI, 0.08–0.24). Increasing the total radiation dose was also inversely associated with the risk of vomiting (OR, 0.97; 95% CI, 0.96–0.99). Other variables, such as age (OR, 0.98; 95% CI, 0.96–1.00), smoking history (OR, 0.72; 95% CI, 0.42–1.24), and chemotherapy history (OR, 0.99; 95% CI, 0.46–2.13), were not statistically significant. Compared with stage I, stage II had an OR of 1.79 (95% CI, 0.94–3.40), stage III had an OR of 2.15 (95% CI, 0.99–4.68), and stage IV had an OR of 1.05 (95% CI, 0.43–2.54) (Table 2).

**Table 2:** Predictive accuracy of the final model in the training dataset.

Predictors	aOR	95%CI
Age	0.98	0.96–1.00
Smoking history	0.72	0.42–1.24
Total radiation dose	0.97	0.96–0.99
Prior chemotherapy	0.99	0.46–2.13
5-HT3receptor antagonist use	0.13	0.08–0.24
StageⅡ of cancer	1.79	0.94–3.40
StageⅢ of cancer	2.15	0.99–4.68
StageⅣ of cancer	1.05	0.43–2.54

ROC-AUC = 0.772 (95%CI: 0.717–0.827).

Brier score = 0.166.

ROC-AUC, receiver operating characteristic—area under the curve; aOR0, adjusted odds ratio; CI, confidence interval; Reference category for cancer stage, StageI

### Receiver operating characteristic-area under the curve (ROC-AUC) and intraclass correlation coefficient in the validation dataset

The selected model, which included age, smoking history, total radiation dose, history of chemotherapy, use of 5-HT3 receptor antagonists, and cancer stage as explanatory variables, demonstrated good predictive performance in the validation dataset. The ROC-AUC was 0.808 (95% CI, 0.763–0.853), indicating a high discriminative ability. Additionally, the Brier score was relatively low (0.135), suggesting low prediction error of the model.

### Calibration of the final model

**Fig. 2:**
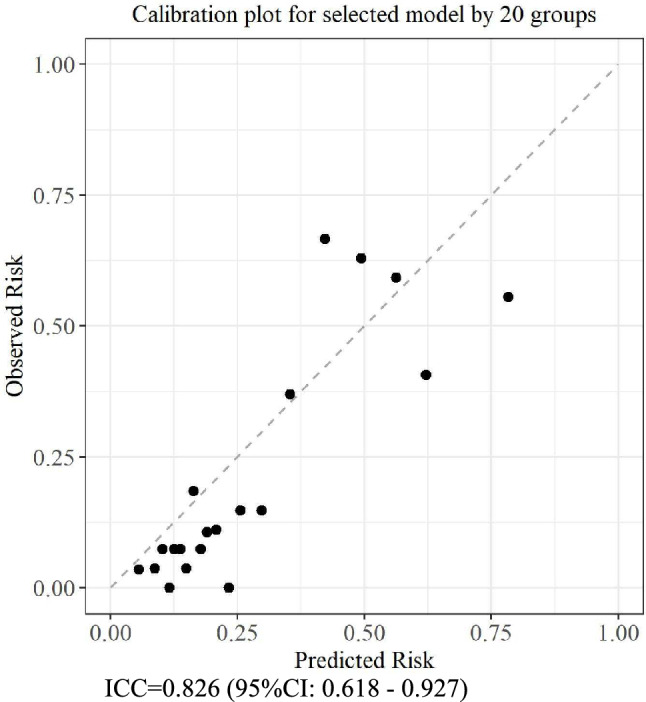
Calibration of the final model. This plot assessed the agreement between the predicted and observed risks by dividing the data into 20 equally sized groups. The diagonal dashed line represents a perfect calibration, where the predicted risk equals the observed risk. ICC, intraclass correlation coefficient; CI, confidence interval.

Figure 2 presents the calibration plot of the selected model, illustrating the relationship between the predicted probabilities and observed risks across 20 equally sized groups. Overall, the predicted risks were generally consistent with the observed risks, indicating a good model calibration. However, in some high-risk groups, the predicted scores tended to exceed the actual observed proportions, suggesting a tendency toward overestimation. However, the predicted values closely followed the ideal 45° line, indicating an acceptable level of agreement between the predicted and actual outcomes (Fig. 2). Despite a tendency for overprediction observed in the calibration plot and a significant Hosmer–Lemeshow test (*p* < 0.0001), the ICC value of 0.826 (95% CI, 0.618–0.927) indicated a considerable degree of agreement.

### Odds ratios and ROC-AUC with the entire dataset and score equations

Subsequently, the optimal model from each selected candidate model pattern was reevaluated using the complete dataset, which integrated both the derivation and validation cohorts. For each final model, the ORs, 95% CIs, ROC-AUCs derived from 200 repetitions of threefold cross-validation, and Brier scores were calculated. The model yielded an ROC-AUC of 0.799 (95% CI, 0.765–0.834) and a Brier score of 0.1, reflecting solid discriminatory power and calibration (Table 3).

**Table 3:** ORs and ROC-AUC with the entire dataset and score equations.

Predictors	aOR	95%CI	*P*
Age	0.99	0.97–1.00	0.080
Smoking history	0.64	0.44–0.93	0.018
Total radiation dose	0.96	0.95–0.97	< 0.001
Prior chemotherapy	0.87	0.48–1.58	0.647
5-HT3receptor antagonist use	0.12	0.08–0.17	< 0.001
Stage II of cancer	1.61	0.98–2.64	0.063
Stage III of cancer	1.94	1.19–3.19	0.008
Stage IV of cancer	1.46	0.78–2.75	0.238

CV ROC-AUC = 0.799 (95%CI: 0.765–0.834).

Brier score = 0.100.

ROC-AUC, receiver operating characteristic—area under the curve; aOR, adjusted odds ratio; CV, cross validation; CI, confidence interval; Reference category for cancer stage, StageI

The predictive model proposed in the present study is defined as follows:

$$\begin{aligned} {\text{P}} = & {\text{1}}/({\text{1}} + {\text{exp}}\left[ {{-}{\text{2}}.{\text{9329}}} \right] + \left[ {{-}0.0{\text{125}}} \right] \times {\text{age}} + \left[ {{-}0.{\text{4439}}} \right] \times {\text{smoking history }} \\ & + \left[ {{-}0.0{\text{384}}} \right] \times {\text{total radiation dose}} + \left[ {{-}0.{\text{1387}}} \right] \times {\text{prior chemotherapy}} + \left[ {{-}{\text{2}}.{\text{1456}}} \right]{\text{ }} \\ & \times {\text{ 5HT3}} + \left[ {0.{\text{4734}}} \right] \times {\text{stageII}} + \left[ {0.{\text{6644}}} \right] \times {\text{stageIII}} + \left[ {0.{\text{38}}0{\text{5}}} \right] \times {\text{stageIV}}) \\ \end{aligned}$$

## Discussion

In the present study, we developed and validated a predictive model for CINV in patients with cervical cancer receiving chemoradiotherapy. To the best of our knowledge, this is the largest multi-institutional retrospective study to date on CINV prediction models for patients with cervical cancer receiving CCRT. Furthermore, the model demonstrated a high predictive performance in the validation dataset, with an ROC-AUC of 0.808. Given the recent reclassification of the emetogenic risk of cervical cancer chemoradiotherapy from moderately emetogenic chemotherapy to HEC in Japan^8^, the need for individualized antiemetic strategies has become increasingly evident. The results of this study suggest that a prediction model tailored to this patient population can facilitate the early identification of high-risk individuals, enabling timely prophylactic interventions and potentially improving patient outcomes.

The high ROC-AUC value observed in this study indicates that our model effectively distinguishes between the high- and low-risk groups for CINV. The risk factors for CINV, including age, female sex, smoking status, radiation dose, and history of chemotherapy, have been previously reported^9–13^. Based on these findings, we incorporated these factors as baseline variables in the predictive model. Furthermore, in collaboration with seven board-certified oncology pharmacists accredited by the Japanese Society of Pharmaceutical Health Care and Sciences, we identified additional factors that were strongly associated with vomiting and had high clinical relevance. These factors, prioritized in the following order—5-HT3 receptor antagonist use and cancer stage—were incorporated to enhance the predictive accuracy of the model^14–16^.

A key strength of our model is its reliance on readily available clinical parameters, which makes it feasible for routine clinical use. Similar approaches have been successfully implemented in other fields, such as the Japan Heart Failure Model for predicting survival in patients with heart failure, demonstrating the value of leveraging existing clinical data for risk stratification^19^. Similarly, our model enables individualized risk assessment and facilitates the selection of more appropriate antiemetic strategies tailored to patient-specific risk profiles. From a clinical perspective, the predictive model proposed in this study represents a practical tool for identifying cervical cancer patients at high risk of CINV prior to the initiation of concurrent chemoradiotherapy. Integration of the model into electronic health record systems could enable automated risk calculation and facilitate early intervention planning. By supporting personalized prophylactic antiemetic strategies, the model has the potential to reduce treatment interruptions, enhance patient comfort, and improve treatment adherence. Moreover, it may aid shared decision-making by enabling clinicians to communicate individualized risk estimates with patients. Future implementation studies are warranted to evaluate the feasibility, clinical utility, and cost-effectiveness of incorporating the model into routine oncology practice. Although specific decision thresholds and scoring algorithms were not defined in the present study, our model lays the foundation for future efforts to derive a clinically actionable risk scoring system. We plan to explore these aspects in subsequent studies aimed at facilitating practical implementation in real-world clinical settings.

A key contribution of this study is the establishment of a predictive model for vomiting events occurring at any point during the full course of concurrent chemoradiotherapy. By extending the observation period to cover the entire treatment duration (typically 6–8 weeks), the model aims to assess the cumulative risk of CINV, beyond the conventional 5-day risk window. Although the risk period for CINV has traditionally been considered to span approximately 5 days from the initiation of chemotherapy, recent studies have reported that CINV may persist beyond 120 h post-administration^13^. Currently, research efforts are underway to develop strategies for managing CINV occurring > 120 h after chemotherapy initiation^20,21^. This personalized approach aligns with the ongoing shift from standardized guideline-based treatments to precision medicine, emphasizing individualized therapeutic decision-making.

According to the OR analysis, olanzapine use was not associated with a reduction in the incidence of CINV. This finding is inconsistent with previous reports demonstrating that the addition of olanzapine to antiemetic regimens for HEC effectively reduces CINV^22–25^. A possible explanation for this is that the study period included the time prior to the revision of the Japanese antiemetic guidelines, during which olanzapine use was relatively rare. In Japan, olanzapine is contraindicated in patients with diabetes, and its use is generally recommended with caution in other countries. Therefore, careful consideration of individual patient characteristics is necessary when prescribing olanzapine.

This study has some limitations. First, the model was developed and validated using a single-country cohort, necessitating external validation in other populations to assess its generalizability. Differences in key variables such as vomiting incidence, olanzapine use, and cancer stage distribution between the training and validation datasets may reflect temporal trends or institutional variability. Although our model demonstrated robust performance despite these imbalances, further validation in independent and prospectively collected cohorts is necessary to confirm its generalizability. Additionally, the significant result of the Hosmer–Lemeshow test (p < 0.0001) should be interpreted with caution. This test is highly sensitive to large sample sizes, and statistical significance does not necessarily indicate poor calibration. Therefore, this result should be considered in conjunction with other calibration metrics, such as the calibration plot and ICC, which supported adequate model calibration. Second, potential confounding factors, such as dietary habits, psychological influences, and genetic predisposition to CINV, were not incorporated into the model. Including these factors in future studies may enhance predictive accuracy. Third, although our model exhibited strong performance as demonstrated by a high ROC-AUC value, its clinical utility remains unconfirmed. In addition, due to the retrospective design of the study, detailed information regarding radiation field and volume was not consistently available, limiting our ability to evaluate their potential association with CINV. The exclusion of patients with non-CCRT-related nausea risk factors may introduce a degree of subjectivity, as these decisions were based on clinical documentation and pharmacist judgment, potentially contributing to selection bias. Finally, as no imputation was conducted, the exclusion of patients with missing data may have introduced selection bias. This limitation should be considered when interpreting the generalizability of the results. Factors such as ease of implementation, clinician adherence, and cost-effectiveness should be evaluated in future studies.

In conclusion, we developed a predictive model for CINV in patients with cervical cancer receiving chemoradiotherapy and demonstrated its high predictive accuracy. This model holds promise for improving supportive cancer care by enabling early risk stratification and personalized antiemetic interventions. Future studies should focus on external validation and clinical implementation to maximize the impact on patient care.

## Supplementary Information

Supplementary Information.

## Data Availability

Due to the nature of this research, participants did not agree to share their data publicly. Data may be available from the corresponding author upon reasonable request.
